# Finger Vein Segmentation from Infrared Images Based on a Modified Separable Mumford Shah Model and Local Entropy Thresholding

**DOI:** 10.1155/2015/868493

**Published:** 2015-05-18

**Authors:** Marios Vlachos, Evangelos Dermatas

**Affiliations:** Department of Electrical & Computer Engineering, Polytechnic Faculty, University of Patras, Rio Campus, 26504 Patras, Greece

## Abstract

A novel method for finger vein pattern extraction from infrared images is presented. This method involves four steps: preprocessing which performs local normalization of the image intensity, image enhancement, image segmentation, and finally postprocessing for image cleaning. In the image enhancement step, an image which will be both smooth and similar to the original is sought. The enhanced image is obtained by minimizing the objective function of a modified separable Mumford Shah Model. Since, this minimization procedure is computationally intensive for large images, a local application of the Mumford Shah Model in small window neighborhoods is proposed. The finger veins are located in concave nonsmooth regions and, so, in order to distinct them from the other tissue parts, all the differences between the smooth neighborhoods, obtained by the local application of the model, and the corresponding windows of the original image are added. After that, veins in the enhanced image have been sufficiently emphasized. Thus, after image enhancement, an accurate segmentation can be obtained readily by a local entropy thresholding method. Finally, the resulted binary image may suffer from some misclassifications and, so, a postprocessing step is performed in order to extract a robust finger vein pattern.

## 1. Introduction

The problem of finger vein extraction from infrared images arises mainly for biometrics purposes but it is also very important for the biomedical research community.

The general structure of a biometric system based on finger veins consists of five main stages: (1)* acquisition* of the infrared images exploiting the absorption of light in near infrared and infrared wavelengths by the different human tissues, (2)* preprocessing* of the acquired images which includes* ROI* (region of interest) extraction, image intensity normalization (in this type of images intensity is usually uneven due to the image acquisition system and may suffer from shading artefacts), and noise reduction, (3)* segmentation or classification* stage in which the preprocessed image divided into two (or more depending on the application) regions associated with veins and surrounding tissues, (4)* postprocessing* of the binary images which delivers the final segmentation result, free of outliers and misclassifications, and finally (5)* matching* of the extracted veins in order to perform the desired person identification/verification procedure. Matching procedure can be applied either directly in the extracted finger vein patterns or in their skeletons, depending on the matching algorithm that has to be used. This general structure described so far involves all the stages that may have such a system but it is worth mentioning that these stages are independent and some of them can be skipped in some applications depending on its specific requirements.

*Related Work*. Several methods which adopt this general architecture have already been presented starting from the pioneering work of Park et al. [1]. In this important research work, an application specific processor for vein pattern extraction and its application to a biometric identification system is proposed. The conventional vein pattern recognition algorithm [1–3] consists of a preprocessing part, applying sequentially an iterative Gaussian low pass, a high pass, and a modified median filter, a recognition part which includes the extraction of the binary veins via local thresholding, and finally the matching between the individual patterns.

An improved vein pattern extraction algorithm is proposed in [4], which compensates the loss of vein patterns in the edge area, gives more enhanced and stabilized vein pattern information, and shows better performance than the existing algorithms. Also, the problem arising from the iterative nature of filtering preprocess is solved by designing a filter that is processed only once, increasing significantly the recognition speed and reducing the hardware complexity. The proposed algorithm is implemented with an* FPGA* device and the false acceptance rate is five times better than the existing algorithm and the recognition speed is measured to be 100 (ms/person).

The problem with conventional hand vascular technology mentioned above is that the vascular pattern is extracted without taking into account its direction. So, there is a loss of vascular connectivity which leads to a degradation of the performance of the verification procedure. An attempt to improve this problem can be found in [5], where a direction based vascular pattern extraction algorithm based on the directional information of vascular patterns is presented for biometric applications. It applies two different filters: row vascular pattern extraction filter for abscissa vascular pattern extraction and column vascular pattern extraction filter for effective extraction of the ordinate vascular patterns. The combined output of both filters produces the final hand vascular patterns. Unlike the conventional hand vascular pattern extraction algorithm, the directional extraction approach prevents loss of the vascular pattern connectivity.

Although the above algorithm considers the directionality of veins, it also assumes that the veins oriented in only two principal directions. In [6, 7] a method for personal identification based on finger vein patterns is presented and evaluated using line tracking starting from various positions. This method allows vein patterns to have an arbitrary direction. Local dark lines are identified and line tracking is executed by moving along the lines pixel by pixel. When a dark line is not detectable, a new tracking operation starts at another position. This procedure executes repeatedly, so the dark lines that tracked multiple times are classified as veins.

Typically, the infrared images of finger veins are low contrast images, due to the light scattering effect. An algorithm for finger vein pattern extraction in infrared images is proposed in [8]. This algorithm embeds all the above issues and proposes novel preprocessing and postprocessing algorithms. Initially, the image is enhanced and the fingerprint lines are removed using* 2D* discrete wavelet filtering. Kernel filtering produces multiple images by rotating the kernel in six different directions, focus on the expected directions of the vein patterns. The maximum of all images is transformed into a binary image. Further improvement is achieved by a two-level morphological process; that is, a majority filter smoothes the contours and removes some of the misclassified isolated pixels, and a reconstruction procedure removes the remaining misclassified regions. The final image is segmented into two regions, the vein and the tissue.

In [9] new issues are considered and a certification system that compares vein images for low cost, high speed, and high precision certification is proposed. The equipment for authentication consists of a near infrared light source and a monochrome* CCD* to produce contrast enhanced images of the subcutaneous veins. The phase correlation and template matching methods are used for classification. Several noise reduction filters, sharpness filters, and histogram manipulations tested for the best effort. As a result, a high certification ratio in this system obtained.

In [10], the theoretical foundation and difficulties of hand vein recognition are introduced at first. Then, the optimum threshold of the segmentation process and the vein lines thinning problem of infrared hand images are deeply studied, followed by the presentation of a novel estimator for the segmentation threshold and an improved conditional thinning method. The method of hand vein image feature extraction based on end points and crossing points is studied initially, and the matching method based on a distance measure is used to match vein images. The matching experiments indicated that this method is efficient in terms of biometric verification.

An efficient automatic method for robust segmentation of finger vessel network and vein pattern extraction from infrared images acquired by a low-cost monochrome or multichannel camera is proposed in [11]. After brightness normalization, the fingerprint lines are eliminated using the 2D dimensional discrete wavelet transformation. A set of twelve directional kernels is constructed, based on a dyadic wavelet transform, for each scale, and is used to enhance the directional properties of veins. From maximum filters' response along scale and direction, a neighbourhood thresholding derives a binary segmented image to produce reliable patterns of finger veins. A postprocessing module is used in case where low quality images are to be segmented. Preliminary evaluation experiments of the proposed method demonstrate a number of advantages, compared to recently published methods.

In the narrow bandwidth of the near infrared spectrum, the light is propagated through human tissue with low absorption rates, but strong scattering effects produce extremely low contrast images. In [12], an algorithm for finger vein segmentation and centerlines extraction in infrared images is presented. Finger veins are detected in pixels with positive divergence of the gradient vector field, while centerlines are extracted in pixels with positive divergence of the normalized gradient vector field estimated at various orientations. The segmentation algorithm has been evaluated on both artificial and real finger infrared images and high segmentation rates are achieved in terms of sensitivity, specificity, and accuracy using manual annotation data obtained by human observers.

A new algorithm for vein matching based on log-polar transform to address problems that occur with the changing of finger position and from differences between imaging devices for current vein matching algorithms is discussed in [13]. The new algorithm first extracts the feature area, which contains enough characteristics for image matching, depending on the structure of the finger vein ridge alignment. It then calculates the degree of similarity between the log-polar transform results of the model image feature areas and the sample image and finally analyzes the result by the degree of similarity and the relationship of relative positions between feature areas. Experiments show that the algorithm is robust for rotating and zooming images of the finger vein.

In [14], four principles (caliber uniformity, node replication, loop splitting, and virtual connection) are proposed, first to simplify the finger vein structure as a binary tree structure. Then a modified binary tree model is proposed based on the binary tree structure. The new model uses the binary tree to describe the relationships between different vein branches and uses a B-spline function to describe the spatial structure of vein branches. Experiments show that this model can quantitatively describe the relationships between, and the spatial structure of, vein branches with little representation error and low storage space requirements.

The method proposed in [15] is rooted in a local binary pattern based method and then inclined to use the best bits only for matching. After presenting the concept of PBBM and the generating algorithm, authors propose the finger vein recognition framework, which consists of preprocessing, feature extraction, and matching. Experimental results show that PBBM achieves not only better performance but also high robustness and reliability. In addition, PBBM can be used as a general framework for binary pattern based recognition.

In [16], a new and robust approach for finger vein ROI localization is introduced, and then a new scheme for effectively improving the visibility of finger vein imageries is proposed. Extensive experiments are conducted to validate this method.

A new method of personal identification based on finger vein recognition is presented in [17]. First, a stable region representing finger vein network is cropped from the image plane of an imaging sensor. A bank of Gabor filters is then used to exploit the finger vein characteristics at different orientations and scales. Based on the filtered image, both local and global finger vein features are extracted to construct a finger vein code (FVCode). Finally, finger vein recognition is implemented using the cosine similarity measure classifier, and a fusion scheme in decision level is adopted to improve the reliability of identification. Experimental results show that this method exhibit an exciting performance in personal identification.

Finally in [18], a finger vein system using the mean curvature that can be used for personal verification is proposed. As a robust extraction method, authors propose the mean curvature method, which views the vein image as a geometric shape and finds the valley-like structures with negative mean curvatures. When the matched pixel ratio is used in matching vein patterns, experimental results show that, while maintaining low complexity, the proposed method achieves 0.25% equal error rate, which is significantly lower than what existing methods can achieve.

However, the finger vein technology, as mentioned above, has also important applications in biomedical field. An initial work for localizing surface veins via near infrared (*NIR*) imaging and structured light ranging is presented in [19]. The eventual goal of the system is to serve as the guidance for a fully automatic (i.e., robotic) catheterization device. The proposed system is based upon near infrared (*NIR*) imaging, which has previously been shown effective in enhancing the visibility of surface veins. The vein regions in the* 2D NIR* images located using standard image processing techniques. An* NIR* line generating* LED* module is used for to implement structured light ranging and construct a* 3D* topographic map of the arm surface. The located veins are mapped to the arm surface to provide a camera registered representation of the arm and veins.

Also in [20, 21], a vein contrast enhancer (*VCE*) has been constructed to make vein access easier by capturing an infrared image of veins, enhancing the contrast using software, and projecting the vein image back onto the skin. The* VCE* also uses software to align the projected image with the original vein and with accuracy of 0.06 mm. Clinical evaluation of earlier monitor based vein enhancement test systems has demonstrated the clinical utility of the infrared imaging technology used in the* VCE*.

Although these methods achieve segmenting the infrared images, the finger vein pattern extraction task is still challenging mainly due to the fact that infrared images suffer from strong noise presence, uneven illumination, and shading, factors that complicate the application of automatic image segmentation techniques. Thus, another way to segment this kind of images is to assume that veins located in thin and concave regions (a reasonable assumption obtained by a careful inspection of the image intensity across the image) of infrared images based on this concept to extract them by optimizing a mathematical model. This can be done by using the Mumford Shah Model which has well-known capabilities in the image processing applications such as image segmentation, restoration, and image inpainting [22, 23]. Thus, in this paper, an analytical solution to a modified Mumford Shah Model minimization problem is derived and a local application of its results, in order to perform fast and accurate finger vein extraction, is proposed.

The remainder of this paper is organized as follows. In Section 2 the experimental device and the image acquisition procedure is presented. In Section 3, a detailed presentation of the finger vein pattern extraction method is given. The experimental results and discussion are included in Section 4. Finally, the most significant conclusions and some directions for future work are presented in the last section of this paper.

## 2. Image Acquisition

**Figure 1:**
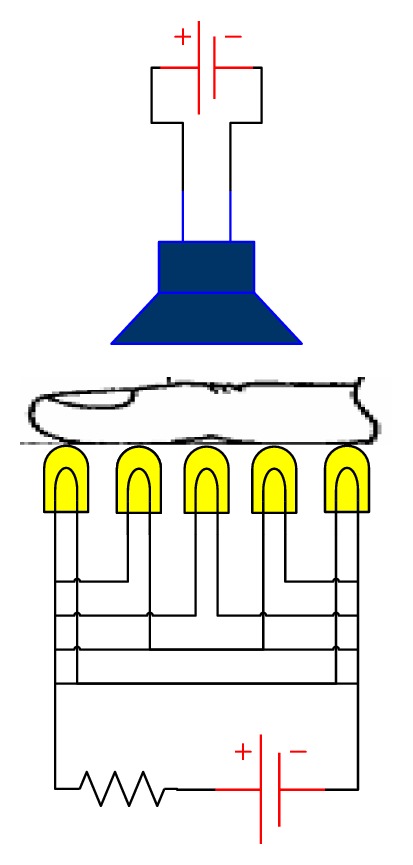
A typical low-cost device used for digital image acquisition of finger infrared images.

A typical hardware used to acquire infrared images consists of a finger probe, an array of infrared leds with adjustable illumination, and a video camera focus on frame, as shown in Figure 1. The finger* ROI* was placed inside the probe, between the open frame and the array of infrared leds light source which consists of a number of leds with adjustable illumination. The finger probe eliminates the influence of the external light sources.

**Figure 2:**
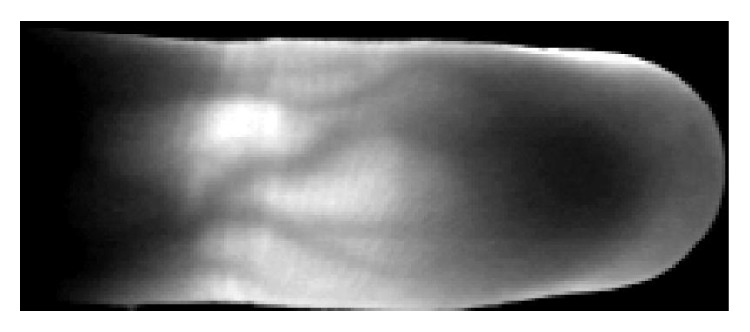
Original image.

The acquired image is produced as a result of several physical phenomena that happen during light propagation through human tissue, that is, absorption, diffusion, and scattering [24]. The great number of substances contained in the human body, the blood dynamics, and the mass transfer phenomena complicates significantly the light transformation effects. Therefore, the solution of the inverse problem, that is, the derivation of the arterial network from the image data, becomes unrealistic. A totally different and popular approach, adopted also in the proposed method, uses several image enhancements, feature extraction, and path reconstruction methods to derive the vein network, based on the fact that substance haemoglobin presents strong absorption in the infrared wavelengths, and therefore the veins appear in the image darker than the other human tissues (Figure 2).

## 3. Detection of Vein Network

**Figure 3:**
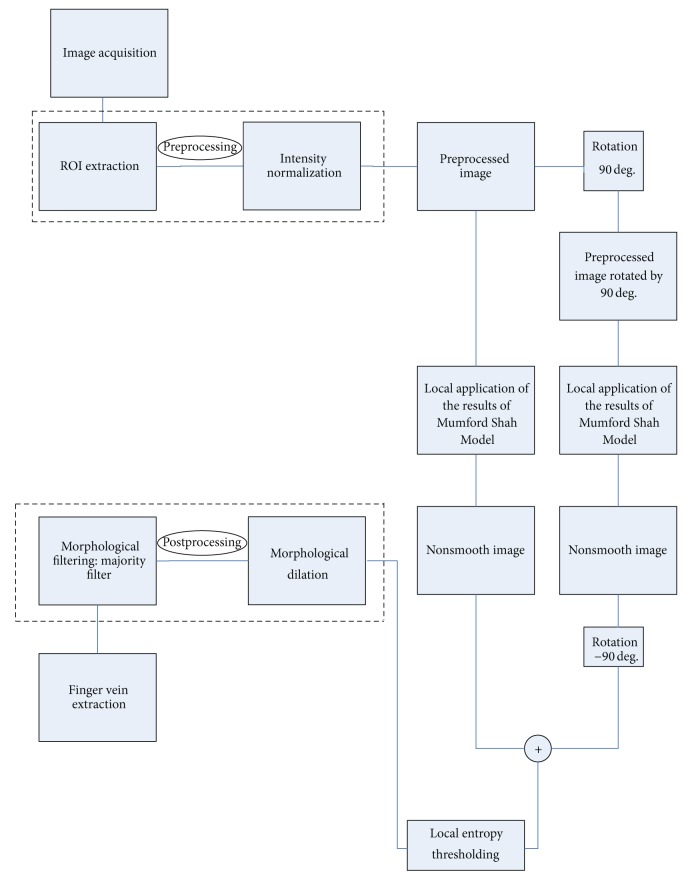
Flowchart of the proposed vein detection method.

In Figure 3, a flowchart of the proposed vein extraction method is given.

### 3.1. Image Preprocessing

#### 3.1.1. *ROI* Extraction

From the original image (Figure 2) a region of interest (*ROI*) is defined based on several statistical properties of the histogram; that is, very low or very high brightness areas are excluded from the* ROI*. In the designed hardware each infrared led has adjustable intensity, giving excellent image quality, minimizing also the variance of the automatic exposure times of the image acquisition system.

**Figure 4:**
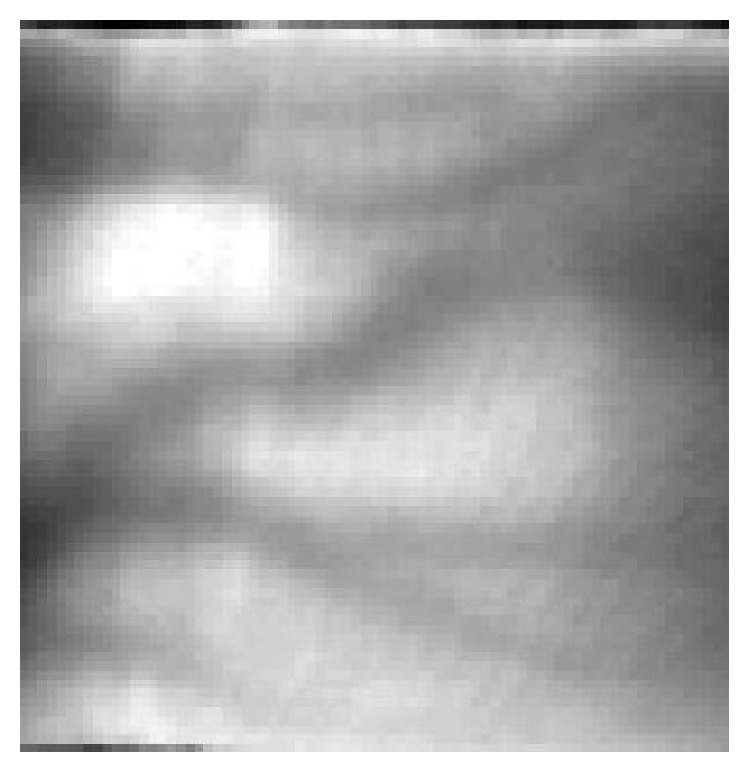
Region of interest extraction (*ROI*).

The acquired image suffers from shading and nonuniform illumination both in left side and in right side of the image. This effect usually influences the performance of automatic image processing methods applied in order to extract the finger vein pattern. Thus,* ROI* extraction is used in order to localize the finger region and to isolate the shading artifacts. In this paper, the method proposed in [25] is adopted for* ROI* extraction. This method is based on the cutoff of regions with shading taking care about the different dimensions of the fingers among each person. Two masks, one for *x* and one for *y* direction, are used to isolate the boundary and localize the effective finger region. A typical* ROI* extracted by the method proposed in [25] is shown in Figure 4.

#### 3.1.2. Brightness Normalization Based on Local Statistical Measures

In general, even in case where led's intensity is adjusted to satisfy several statistical properties, in few areas of the acquired image unsatisfactory illumination or strong noise distortions are met. Therefore an image normalization procedure is applied to restore partially the desirable characteristics.

The proposed local normalization procedure unifies the local mean and variance of the* ROI*, especially useful technique for correcting nonuniform illumination or shading artifacts, using a linear transformation scheme applied on pixels' brightness,

(1) $$\begin{array}{c} u_{0}\left(x,y\right)=\frac{r\left(x,y\right)-m_{r}\left(x,y\right)}{\sigma_{r}\left(x,y\right)}, \end{array}$$

where *r*(*x*, *y*) is the brightness of the original* ROI* image at pixel (*x*, *y*), *m*_*r*_(*x*, *y*) is the brightness local mean, *σ*_*r*_(*x*, *y*) is the corresponding local standard deviation, and *u*_0_(*x*, *y*) is the normalized image. The estimation of local mean and standard deviation is performed inside small window neighborhoods by averaging pixel intensities, a process also known as spatial smoothing.

### 3.2. Minimization of the Mumford Shah Model

The human veins in finger are significantly thinner than the darker structures observed in typical infrared images, as shown in Figure 2. Multiple scattering of the propagated photons reduces significantly the contrast, eliminates the tiny veins, and increases the transition regions between the vein and the surrounding tissue. The “fog” effect hides the vein structures in concave regions of the* ROI*. This assumption could be verified by observing the cross section profile of the veins which is Gaussian-like, as claimed in [26]. The aim of the proposed system is to focus on the concave regions enhancement, based on several connectivity properties in order to simply separate them from the rest tissue by a local entropy thresholding technique.

The concave regions are regions in the image domain where the second order derivative is positive. Direct estimation of the derivatives in digital images is an ill-posed problem due to noise presence and the variations in illumination. Instead of seeking regions which have positive second order derivatives, the minimization of an objective function similar to the objective function of the Mumford Shah Model [27] is proposed. The objective of the Mumford Shah Model is to estimate a smooth function *u* such that it is similar to the original image *u*_0_. The equivalent mathematical expression leads to the minimization problem of the following objective function:

(2) $$\begin{array}{c} J\left(u\right)=\int_{\Omega }{\left|\nabla u\right|}^{2}dx+\lambda \int_{\Omega }{\left|u-u_{0}\right|}^{2}dx, \end{array}$$

where *Ω* is the image domain and *λ* is a user defined parameter. The minimization of this function is computationally intensive and can be performed by the method proposed by Chan and Shen [22]. This method belongs to the category of segmentation methods which use partial differential equations (*PDE*) and it is iterative. Instead of deriving the global minimum of (2), in this paper a close form solution of a discrete objective function (3) similar to the objective function of the Mumford Shah Model is proposed by processing small rectangular areas. The proposed solution accelerates significantly the processing time and outperforms the classical approach [22]. As a result, fast and accurate extraction of the finger veins is obtained.

Assuming, without loss of generality, that the original image has *N* rows and *N* columns (however the image can have different dimensions along the two axes), the function *J*(*u*) in the discrete space is defined as follows:

(3) $$\begin{array}{c} J\left(u\right)=\frac{1}{2}\cdot \overset{N}{\underset{i=1}{\sum }}\;\overset{N}{\underset{j=1}{\sum }}{\left|\nabla u\right|}^{2}+\frac{\lambda }{2}\cdot \overset{N}{\underset{i=1}{\sum }}\;\overset{N}{\underset{j=1}{\sum }}{\left|u-u_{0}\right|}^{2}, \end{array}$$

where ∇*u* is the gradient of the image *u*(·, ·) and can be written as

(4) $$\begin{array}{c} {\left|\nabla u\right|}^{2}={\left(\frac{\partial u}{\partial x}\right)}^{2}+{\left(\frac{\partial u}{\partial y}\right)}^{2}. \end{array}$$

If the partial derivatives in (4) are approximated using local differences,

(5) $$\begin{array}{c} \frac{\partial u}{\partial x}=u\left(x+1,y\right)-u\left(x,y\right),\\ \frac{\partial u}{\partial y}=u\left(x,y+1\right)-u\left(x,y\right), \end{array}$$

and (4) is substituted in (3) the following formula is obtained:

(6) Ju=12·∑x=1N−1 ∑y=1N−1ux+1,y−ux,y2     +ux,y+1−ux,y2+λ2·∑x=1N−1 ∑y=1N−1ux,y−u0x,y2.

The minimum of this objective function (6) regarding *u*(·, ·) can be derived in a close form by differentiating the second order, positively defined function:

(7) ∂J(u)∂u=12·∑x=1N−1 ∑x=1N−1∂∂uux+1,y−ux,y2          +ux,y+1−ux,y2    +λ2·∑x=1N−1 ∑x=1N−1∂∂uux,y−u0x,y2=0⟹(λ+4)·u(x,y)−u(x+1,y)   −u(x,y+1)−u(x,y−1)−u(x−1,y)  =λ·u0(x,y), ∀x,y∈1,N−1.

As it can easily be observed, (7) is applied for pixels ∀(*x*, *y*)∈[1, *N* − 1] in order to avoid the boundary treatment problem for the finite difference scheme. Thus, the necessary image points are available for this type of approximation scheme. Obviously, this approach has a truncation error but, as proved experimentally, it has negligible impact in the performance of the proposed method.

Equation (7) can be rewritten in matrix form as

(8) λ+4−100·−1·−1λ+4−10··−10−1λ+4−1···00−1····−1·····−1·−1···−1λ+400−10·0−100−1·0−1λ+4  ·u1,1u1,2·u1,Nu2,1u2,2·u2,N·uN,1·uN,N=λ·u01,1u01,2·u01,Nu02,1u02,2·u02,N·u0N,1·u0N,N,

which is in the form *A* · *x* = *λ* · *b*. *A* is a sparse Hermitian matrix that depends only on parameter *λ* coefficients and size (*N* ^2^ × *N* ^2^), *x* is the vector of unknown image *u*(·, ·)  (*N* ^2^ × 1), and *b* is the vector of the original image *u*_0_(·, ·)  (*N* ^2^ × 1). If the matrix *A* is invertible, the brightness of the unknown image is derived from the solution of the system of linear equations:

(9) $$\begin{array}{c} x=A^{-1}\cdot \left(\lambda \cdot b\right). \end{array}$$

The matrix is invertible if the determinant is nonzero (see Appendix—Lemmas A.1 and A.2 and Theorem A.3).

### 3.3. Modified Mumford Shah Model

From the above analysis, a sparse Hermitian matrix *A* has been arisen. This matrix, as it is evident from (8), has the value *λ* + 4 in its central diagonal, the value −1 in the next up and down diagonal, and the value −1 *N* positions before and after the central diagonal. Thus, it is a block tridiagonal matrix which can be inverted using an iterative algorithm such as one presented in [28]. Instead of using this computationally exhaustive algorithm, the fact that the above form of matrix *A* can be obtained from two independent minimizations is exploited: one for the second order partial derivative in the *x*-axis and one for the second directional derivative in the *y*-axis. Thus, the following two objective functions have to be minimized with respect to *u*(·, ·):

(10) $$\begin{array}{c} J\left(u\right)=\frac{1}{2}\cdot \overset{N}{\underset{i=1}{\sum }}\;\overset{N}{\underset{j=1}{\sum }}{\left(\frac{\partial u}{\partial x}\right)}^{2}+\frac{\lambda }{2}\cdot \overset{N}{\underset{i=1}{\sum }}\;\overset{N}{\underset{j=1}{\sum }}{\left|u-u_{0}\right|}^{2},\\ J\left(u\right)=\frac{1}{2}\cdot \overset{N}{\underset{i=1}{\sum }}\;\overset{N}{\underset{j=1}{\sum }}{\left(\frac{\partial u}{\partial y}\right)}^{2}+\frac{\lambda }{2}\cdot \overset{N}{\underset{i=1}{\sum }}\;\overset{N}{\underset{j=1}{\sum }}{\left|u-u_{0}\right|}^{2}. \end{array}$$

These minimization problems (10) led to the following set of equations:

(11) Ju=12·∑x=1N−1 ∑y=1N−1ux+1,y−ux,y2+λ2·∑x=1N−1 ∑y=1N−1ux,y−u0x,y2,Ju=12·∑x=1N−1 ∑y=1N−1ux,y+1−ux,y2+λ2·∑x=1N−1 ∑y=1N−1ux,y−u0x,y2.

By differentiating above two equations the following formulas are obtained:

(12) ∂J(u)∂u=12·∑x=1N−1 ∑x=1N−1∂∂uux+1,y−ux,y2    +λ2·∑x=1N−1 ∑x=1N−1∂∂uux,y−u0x,y2=0⟹(λ+2)·u(x,y)−u(x+1,y)−u(x−1,y)   =λ·u0(x,y), ∀x,y∈1,N−1,

(13) ∂J(u)∂u=12·∑x=1N−1 ∑x=1N−1∂∂uux,y+1−ux,y2    +λ2·∑x=1N−1 ∑x=1N−1∂∂uux,y−u0x,y2=0⟹(λ+2)·u(x,y)−u(x,y+1)−u(x,y−1)   =λ·u0(x,y), ∀x,y∈1,N−1.

Equations (12), (13) can be written in matrix form as

(14) λ+2000·−1·0λ+200··−100λ+20···000····−1·····0·−1···0λ+200−10·0000−1·00λ+2  ·u1,1u1,2·u1,Nu2,1u2,2·u2,N·uN,1·uN,N=λ·u01,1u01,2·u01,Nu02,1u02,2·u02,N·u0N,1·u0N,N,

(15) λ+2−100···−1λ+2−10···0−1λ+2−1···00−1··········−1·····−1λ+200·0·0−100··0−1λ+2  ·u1,1u1,2·u1,Nu2,1u2,2·u2,N·uN,1·uN,N=λ·u01,1u01,2·u01,Nu02,1u02,2·u02,N·u0N,1·u0N,N.

From (14) and (15) it can be observed that in (14) the matrix *A* has the value *λ* + 2 in its central diagonal and the value −1 *N* positions before and after the central diagonal while in (15) the matrix *A* has the value *λ* + 2 in its central diagonal and the value −1 in the next up and down diagonal. Thus, the combination of the two matrices can give us the same results (see Appendix—Statement) as the matrix *A* presented in (8). By exploiting this fact we can use only the matrix *A* obtained by (15) instead of using the matrix *A* obtained from (8). The advantage of this approach is that in this case the inversion of a symmetric matrix (15) is required which can be obtained analytically (see Appendix) and without the computationally exhaustive approaches adopted in [28–34]. In the sequel, (9) is applied one time for the original image *u*_0_(·, ·) and one for its rotated version by 90 degrees. This is done because two objective functions (one in the *x* direction and one in the *y* direction) have to be minimized. The enhanced image is derived by the addition (after normalization) of the two independent results (after the rotation of the second image *u*(·, ·) by −90 degrees in order to have a meaningful result):

(16) $$\begin{array}{c} x_{\left(0\text{degrees}\right)}=A^{-1}\cdot \left(\lambda \cdot b_{\left(0\text{degrees}\right)}\right), \end{array}$$

(17) $$\begin{array}{c} x_{\left(90\text{degrees}\right)}=A^{-1}\cdot \left(\lambda \cdot b_{\left(90\text{degrees}\right)}\right), \end{array}$$

(18) $$\begin{array}{c} u=x_{\left(0\text{degrees}\right)}+x_{\left(90\text{degrees}\right)}, \end{array}$$

where *b*_(0degrees)_, *b*_(90degrees)_ are the original image (rearranged as vector) and its rotated version (also rearranged as vector) by 90 degrees, respectively, *A* ^−1^ is the inverse of the matrix presented in (15), and *x*_(0degrees)_, *x*_(90degrees)_ are the resulting smooth image and its rotation by 90 degrees version. Finally, *u* is their sum after the rotation of *x*_(90degrees)_ by –90 degrees in order to have a meaningful result.

The determinant of the matrix *A* must be nonzero in order to be invertible. This is proved in Lemmas A.1 and A.2 (see Appendix).

In practice, even in the case of small images, that is, 100 × 100 pixels, the inversion of a sparse matrix *A* of 10000 × 10000 is required. This is obvious of too high computational cost. An effective reduction of the matrix *A* dimensionality can be achieved using subimages, that is, multiple solutions of (13), using only a small number of neighbor pixels. In this case the number of linear equations depends on the size of the chosen window.

From the optimization criterion, the new image *u* is a smooth image similar to the original *u*_0_. As a result of the image transformation method the veins are located in concave nonsmooth regions, so the veins network is enhanced in the nonsmooth image *u* − *u*_0_.

In order to obtain the image *u* − *u*_0_ the following process is applied. Initially a window of size *MxM* is selected and the corresponding matrix *A*  (*M* ^2^ × *M* ^2^) and its inverse inv*A*  (*M* ^2^ × *M* ^2^) are estimated. Then, the sliding window is moved along each pixel of the original image and the difference between the pixel values (of the image *u* inside the window) obtained by (16) or (17) and the pixel values of the original image *u*_0_ inside the window is computed. In the next step all these window differences are added and the image *u* − *u*_0_ is obtained by keeping the central *N* × *N* part of the result (where *N* × *N* is the size of the original image).

The result of this process is the nonsmooth image *u* − *u*_0_. In this image the veins located in concave regions and for this reason a local entropy thresholding technique is applied in order to segment the nonsmooth image in concave (veins) and nonconcave (other tissue parts) regions.

This procedure is repeated for two times: one time for the original image and one for its rotation version by 90 degrees. The two nonsmooth images are added in order to obtain the final enhanced image *u*  (18).

To conclude, the concept of the proposed method originates from the continuous Mumford Shah Model. However, in this paper, a modified separable discrete model (11) is proposed and the transition between continuous and discrete space is not straightforward. Equation (3) is used only for indicating the conceptual similarity between the proposed discrete model and the continuous Mumford Shah Model because it is deemed that it is not fair to present the proposed model as an entirely novel model. Thus, the actual minimization is performed in models presented in (6) and (11) for the modified discrete and the modified separable discrete model, respectively.

### 3.4. Local Entropy Thresholding

Among the various existing methods used to automatically define the threshold for segmentation, the local entropy thresholding is selected, which has been successfully used in [35], because pixel intensities are not independent and this efficient entropy based thresholding takes into account the spatial distribution of intensities. It is based on the estimation of the cooccurrence matrix of the image *u* − *u*_0_ which is a measure of the transition of intensities between adjacent pixels. Specifically, a local entropy thresholding technique, described in [36], is implemented which can preserve the structure details of an image. Two images with identical histograms but different spatial distribution will result in different entropy (also different threshold values).

In a correction note N. R. Pal and S. K. Pal [37] propose two modifications to improve the results of blood vessel extraction that are essential to the performance of image registration. These modifications were adopted also in our study because they experimentally proved superior to [36] (see Appendix—Local Entropy Thresholding).

### 3.5. Postprocessing

#### 3.5.1. Morphological Dilation

The resulting binary image tends to suffer from some misclassifications (outliers). In order to have a robust segmentation in this postprocessing substep a morphological dilation [38] with a line structuring element oriented along the *x*-axis and elongated *Y* pixels are performed. The output of this step is an image with less outliers but with still evident misclassifications. The final morphological filtering substep that follows gives us the desired robust finger vein pattern.

#### 3.5.2. Morphological Filtering

In this substep the image is postprocessed by applying iteratively a morphological filter called majority [38]. This filter sets a pixel to 1 if five or more pixels in its 3-by-3 neighbourhood has the value 1; otherwise, it sets the pixel to 0. This filter is applied iteratively until the output image remains unchanged. This application clears the image from small misclassified regions which appears due to the presence of noise and smoothes the contours.

## 4. Experimental Results

### 4.1. Real Image Database

The original image was acquired under infrared light using an inexpensive* CCD* camera. The finger was placed between the camera and the light source which consists of a row of infrared leds (five elements) with adjustable illumination. The intensity of the leds adjusted as far as the illumination of the image was good enough. However, the problem of acquisition of infrared images is not a trivial task. The phenomena which were involved in the transmission of light inside the human tissue are very complicated. This fact does not permit us to acquire a sufficient number of images in order to construct an infrared finger image database and to release it in the research community for evaluation and comparison purposes. Our future work is mainly focused on the direction of improvement of the image acquisition system.

An excellent image illumination is not a strict requirement because the good performance of the proposed method remains also under adverse illumination conditions. Due to the fact that haemoglobin has strong absorption in the infrared light the veins are shown in the image darker than the other human tissues. So, the goal of our study is to extract these dark regions, corresponding to veins, from the background, corresponding to the other human parts (tissue). The original image which was acquired as described above is shown in Figure 2.

In this section the results of the application of our method and the methods proposed in [6, 7, 11, 12] are presented. The qualitative evaluation of methods performed in the* ROI* image is shown in Figure 4 and in the original image shown in Figure 2.

### 4.2. Proposed Method

For both images a window neighbourhood of size 9 × 9 is used which results in an 81 × 81 matrix *A* which can be inverted very quickly. The selection of the parameter *λ* does not affect the performance of our method and thus it is arbitrarily selected as *λ* = 1. Parameter *λ* is a weighting factor between the two terms of (2). It accounts for the degree of similarity between the original image and the estimated image. Extensive experiments are conducted in order to justify the selection of parameter *λ*. During the experiments the evaluation rates did not vary very much by changing the parameter *λ* from 0.1 to 1. The average divergence on the results was almost 2%. For the threshold computation the modified local entropy thresholding technique is used as described in the correction note [37]. In postprocessing step, a line structuring element with *Y* = 5 pixels in length and oriented in the *x*-axis is employed.

**Figure 5:**
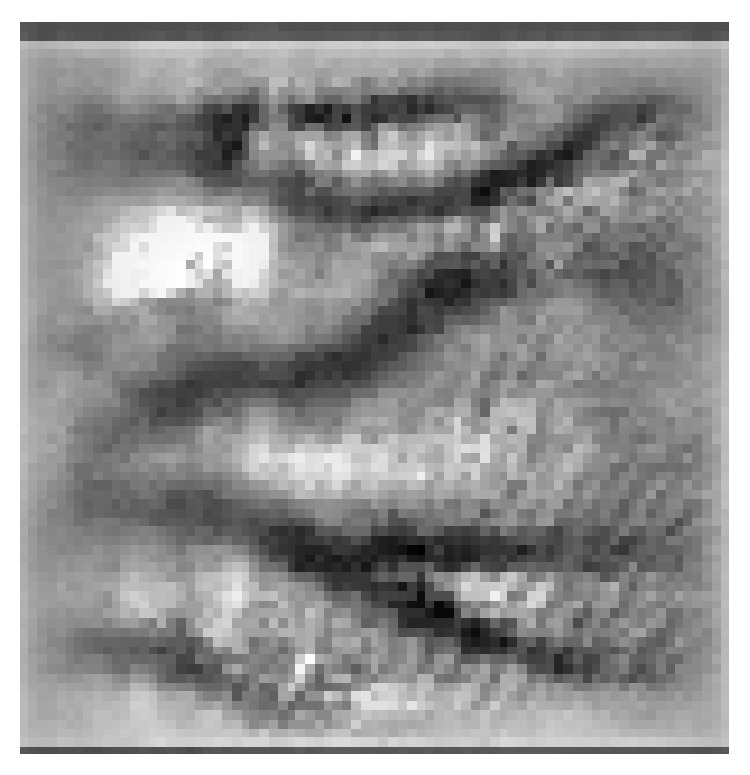
Nonsmooth image.

**Figure 6:**
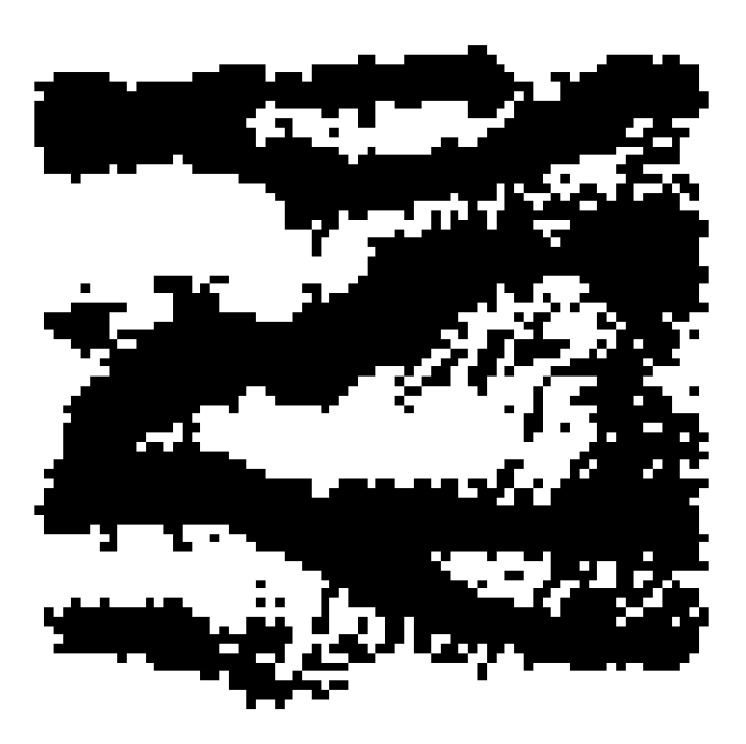
Concave (black) and nonconcave regions (white).

**Figure 7:**
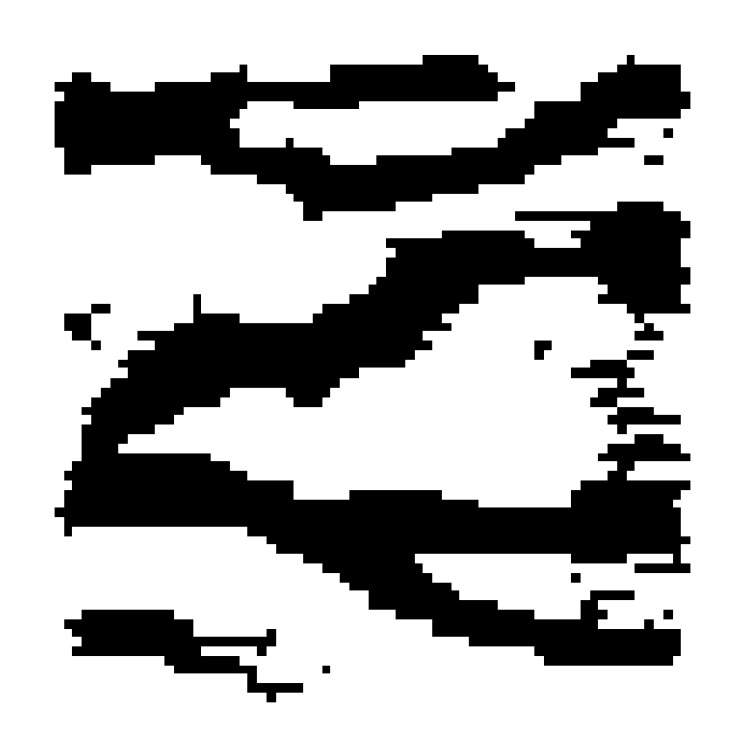
Morphological dilation.

**Figure 8:**
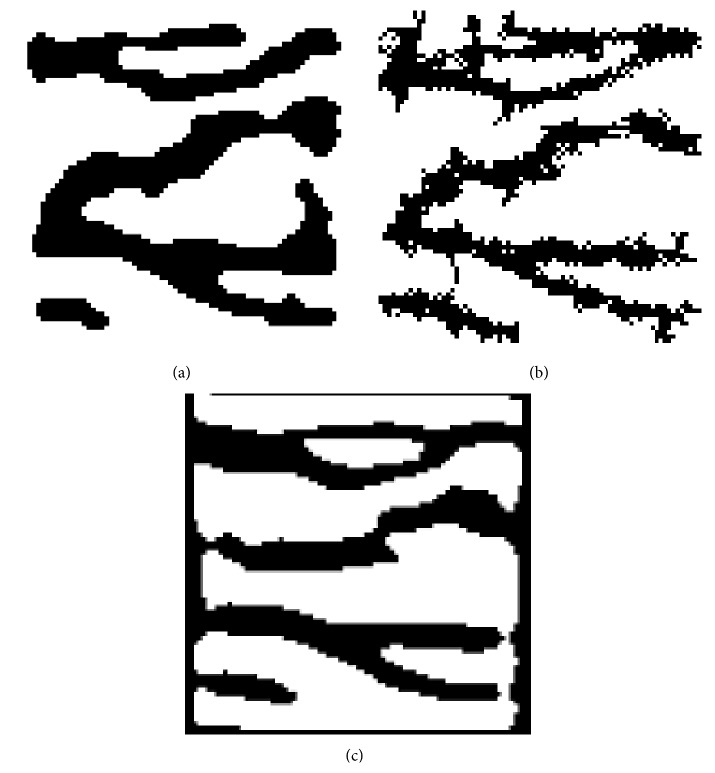
(a) Morphological (majority) filtering: extracted finger vein pattern using the proposed method, (b) extracted finger vein pattern using method [6, 7], and (c) extracted finger vein pattern using method [11].

Figures 5–8 present the results of the application of the proposed method in the* ROI* image of Figure 4.  Figure 5 shows the nonsmooth image obtained after the application of the modified separable Mumford Shah Model in both 0 and 90 degrees direction and the addition of the results.  Figure 6 shows the detection, with the help of the modified local entropy thresholding, of the concave regions of the image, where the veins tend to locate. In this binary image, concave regions (candidate pixels to be detected as veins) are shown in black while other tissue parts shown in white.  Figure 7 shows the binary image after the application of the morphological dilation substep and, finally, the image in Figure 8(a) shows the extracted finger vein pattern obtained after the final morphological filtering substep.

**Figure 9:**
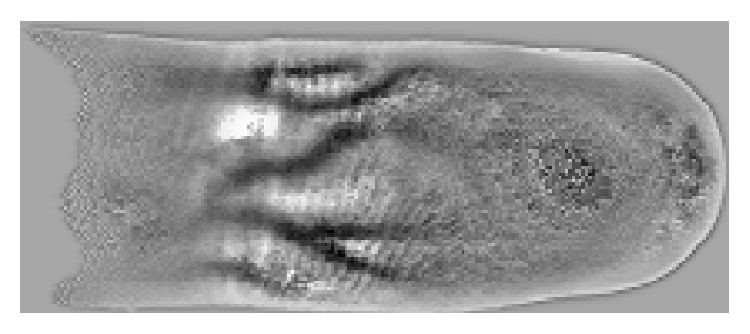
Nonsmooth image.

**Figure 10:**
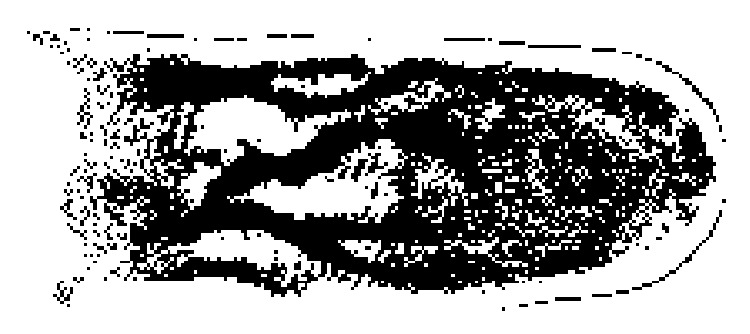
Concave (black) and nonconcave regions (white).

**Figure 11:**
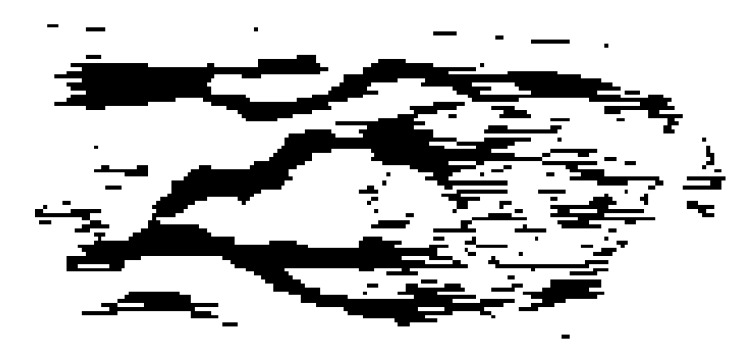
Morphological dilation.

**Figure 12:**
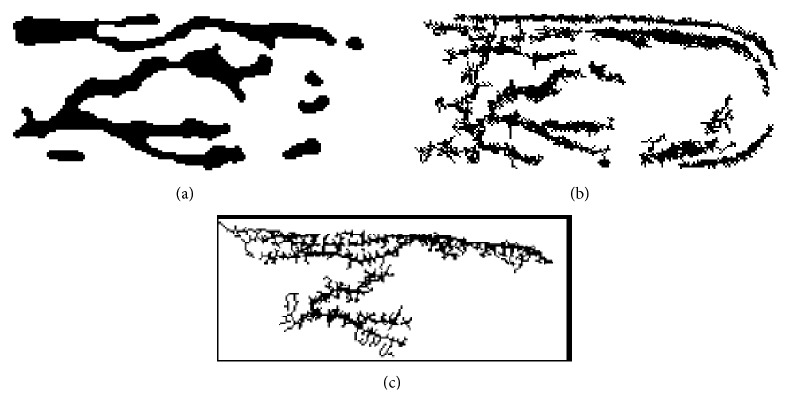
(a) Morphological (majority) filtering: extracted finger vein pattern using the proposed method, (b) extracted finger vein pattern using method [6, 7], and (c) extracted finger vein pattern using method [12].

Moreover, the results of the application of our method in the original image of Figure 2 (before* ROI* extraction) are presented.  Figure 9 shows the nonsmooth image obtained after the application of the modified separable Mumford Shah Model in both 0 and 90 degrees directions and the addition of the results.  Figure 10 shows the detection, with the help of the modified local entropy thresholding, of the concave regions of image. In this binary image, concave regions (candidate pixels to be detected as veins) are shown in black while other tissue parts shown in white.  Figure 11 shows the binary image after the application of the morphological dilation substep and finally Figure 12(a) shows the extracted finger vein pattern obtained after the final morphological filtering substep.

### 4.3. Method [6, 7]

As mentioned, the proposed method is compared with our implementation of the method presented in [6, 7]. The repeated line tracking method requires some parameter tuning in order to run and the robustness of the extracted finger vein pattern is strongly affected by the number of repetitions. The parameters used in the experiments are *N* = 10000 for the number of iterations, *p*_*lr*_ = 50 and *p*_*ud*_ = 25 for the probabilities of selecting the three neighboring pixels in the horizontal or vertical direction, respectively, *W* = 9 for the width of the profiles, and *r* = 1 for the distance between the testing pixel and the cross section. In order to perform a fair comparison between method [6, 7] and the proposed method the same morphological postprocessing step is used in all experiments.

### 4.4. Other Methods

Moreover, the proposed method is also compared with more recent methods such as those presented in [11, 12], briefly described in the Related Work section, due to the fact that these methods have been applied in the same real and artificial image databases and thus the comparison is reasonable.

For purposes of comparison the results of the application of methods [6, 7, 11, 12] in original and ROI image are presented in the same figures as the results of the proposed method. Thus, Figure 8(b) shows the extracted finger vein pattern produced after the application of the line tracking method in the* ROI* image of Figures 4 and 8(c) shows the extracted finger vein pattern produced after the application of the method [11] in the* ROI* image of Figure 4 while Figure 12(b) shows the extracted finger vein pattern produced after the application of the line tracking method in the image of Figures 2 and 12(c) shows the extracted finger vein pattern produced after the application of the method [12] in the image of Figure 2.

Observing Figures 8 and 12, it is obvious that no safe conclusions regarding the performance of all methods can be conducted by visual inspection of the images. Instead, a comparison based on widely known measures should be done. Unfortunately, there is not a publicly available database existing that can be used for evaluation and comparison purposes between various methods. However, an artificial finger image database is constructed in this study in order to evaluate the proposed method and to compare it with the methods presented in [6, 7, 11, 12]. It is worth mentioning that the results presented for the method [6, 7] are produced by the application of our implementation to images since the code of the method is not publicly available by the authors.

### 4.5. Artificial Image Database

A quantitative evaluation of the proposed method in real infrared images is difficult due to the absence of manual segmentation data. The extremely low contrast images increase the disagreement of human annotation. Therefore, the proposed method is evaluated using a small set of images, each one created by the weighed sum of two artificial images. The first image is constructed using an artificial vein-like network. This network consists of connected lines of different widths with junctions and bifurcations and multiple low pass filtering to simulate the blurriness of the edges which is apparent to the real images due to the blood flow and scattering effects. The second artificial image is used to simulate the nonuniform image background of real infrared images created by applying an iterative spatial low pass Gaussian filter with a large window size to the original infrared image.

### 4.6. Evaluation Rates

In the finger vein segmentation process, each pixel is classified as tissue (nonvein) or vein. Consequently, there are four events, true positive (TP) and true negative (TN) when a pixel is correctly segmented as vein or nonvein and two misclassifications: a false negative (FN) appears when a pixel in a vein is segmented in the nonvein area, and a false positive (FP) when a nonvein pixel is segmented as a vein pixel.

Two widely known statistical measures are used for method evaluation: sensitivity and specificity, which are used to evaluate the performance of the binary segmentation outcome. The sensitivity is a normalized measure of true positives, while specificity measures the proportion of true negatives:

(19) $$\begin{array}{c} \text{sensitivity}=\frac{\text{TP}}{\text{TP}+\text{FN}},\\ \text{specificity}=\frac{\text{TN}}{\text{TN}+\text{FP}}. \end{array}$$

Usually, there is a tradeoff between two measures. Finally, the accuracy of the binary classification is defined by

(20) $$\begin{array}{c} \text{accuracy}=\frac{\text{TP}+\text{TN}}{P+N}, \end{array}$$

where *P* and *N* represent the total number of positive (vein) and negative (nonvein) pixels in the segmentation process and are the degree of conformity between the estimated binary classification and the ground truth obtained through a manual segmentation. Thus, the accuracy is strongly related to the segmentation quality and for this reason it is used to evaluate and compare different methods.

### 4.7. Evaluation Results

The proposed method and the methods [6, 7, 11, 12] are evaluated quantitatively on the artificial image database. Each image of the set is constructed according to the above procedure. The evaluation is performed using the widely known statistical measures of sensitivity, specificity, and accuracy.

**Figure 13:**
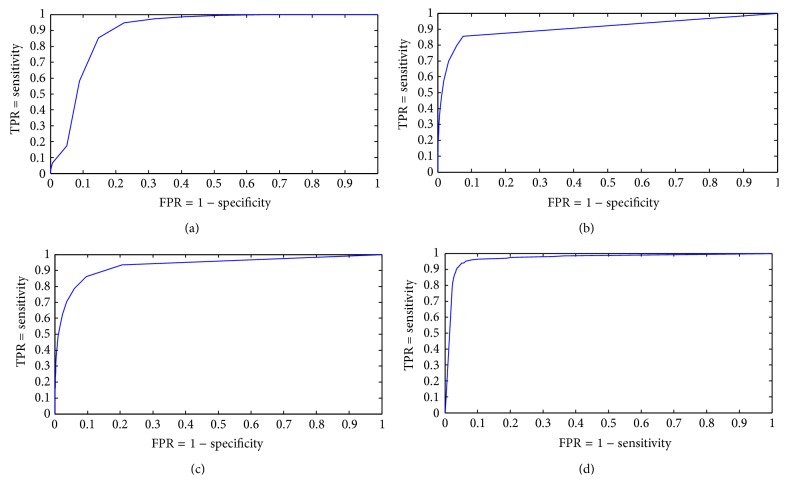
(a)* ROC* curve of the proposed method produced by varying the local segmentation threshold, (b)* ROC* curve of the method proposed in [6, 7] produced by varying the local segmentation threshold, (c)* ROC* curve of the method proposed in [11] produced by varying the local segmentation threshold, and (d)* ROC* curve of the method proposed in [12] produced by varying the value of the threshold parameter *φ*_3_.

Table 1 shows the mean sensitivity, specificity, and accuracy of the proposed method and the methods [6, 7, 11] (without preprocessing/postprocessing) and [12] on the artificial finger image database, while Figure 13(a) shows the* ROC* curve of the proposed method produced by varying the local segmentation threshold and estimating the corresponding measures, Figure 13(b) shows the* ROC* curve of the method [6, 7] produced by varying the local segmentation threshold and estimating the corresponding measures, Figure 13(c) shows the* ROC* curve of the method [11] produced by varying the local segmentation threshold and estimating the corresponding measures, and Figure 13(d) shows the* ROC* curve of the method [12] produced by varying the value of the threshold parameter *φ*_3_ and estimating the corresponding measures.

**Figure 14:**
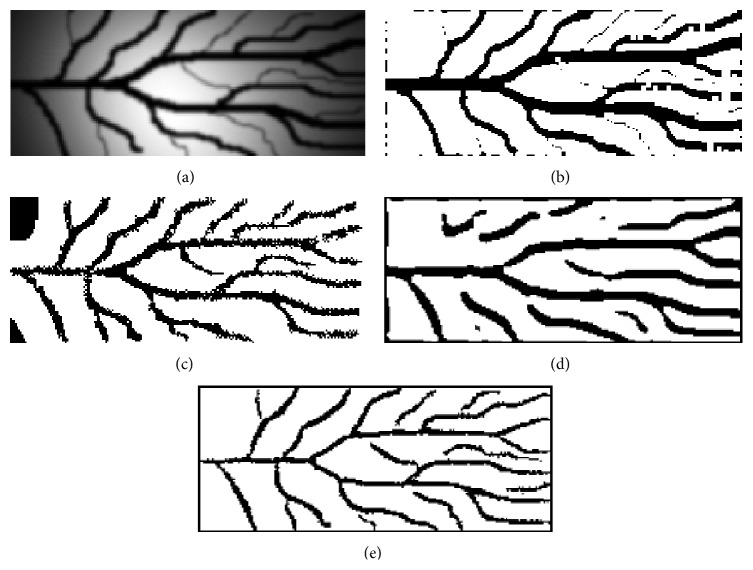
(a) Artificial infrared finger image, (b) extracted vein pattern using the proposed method, (c) extracted vein pattern using the method [6, 7], (d) extracted vein pattern using the method [11], and (e) extracted vein pattern using the method [12].

Figure 14(a) shows the first image of the artificial image database used for the evaluation of the proposed method and the corresponding results of the segmentation for the proposed method (Figure 14(b)) and for the methods [6, 7] (Figure 14(c)), [11] (Figure 14(d)), and [12] (Figure 14(e)), respectively.

By observing the results presented in Figure 14 and in Table 1, it seems that the proposed method performs better than the methods presented in [6, 7, 11] in artificial finger image database in terms of sensitivity, specificity, and accuracy and has comparable performance with the method presented in [12] using the same evaluation criteria. In addition, the visual inspection of images shown in Figure 12 leads to the conclusion that the proposed method performs extremely better than the method [12] in real finger images. Thus, the proposed method, unlike the others compared, robustly extracts the finger vein network and preserves its connectivity against various conditions (shading, intensity variations, and noise distortion) both to real and to artificial finger images.

Apart from evaluating and comparing the proposed method to real and artificial finger vein images authors present also the results of the application of the proposed method and method [6, 7] in artificially distorted images using different types of noise and different levels of distortion. The study of the effect of noise led to an adaptation and a modification of the proposed method in order to robustly perform under extreme conditions. These conditions, although they seem to be unrealistic, may be produced by a low quality acquisition system and its noncareful setup.

**Figure 15:**
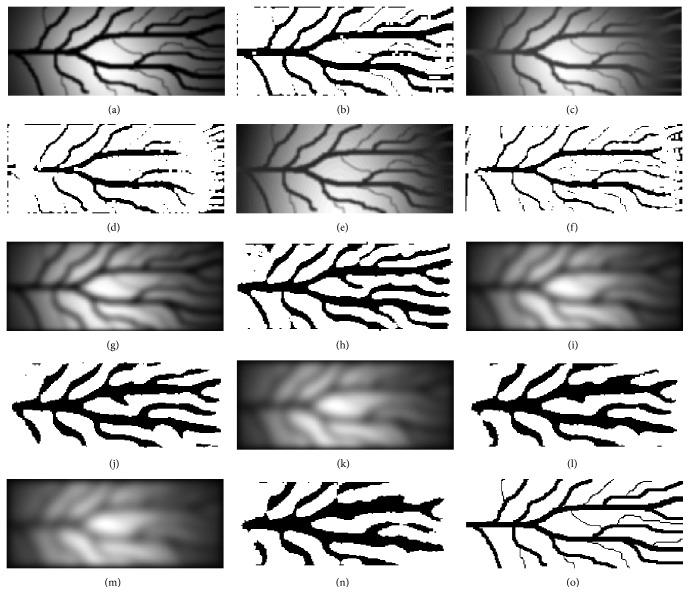
(a, c, e, g, i, k, and m) Artificial infrared images with different level of distortion, (b, d, f, h, j, l, and n) corresponding finger vein patterns extracted using the proposed method, and (o) manual segmentation (ground truth).

**Figure 16:**
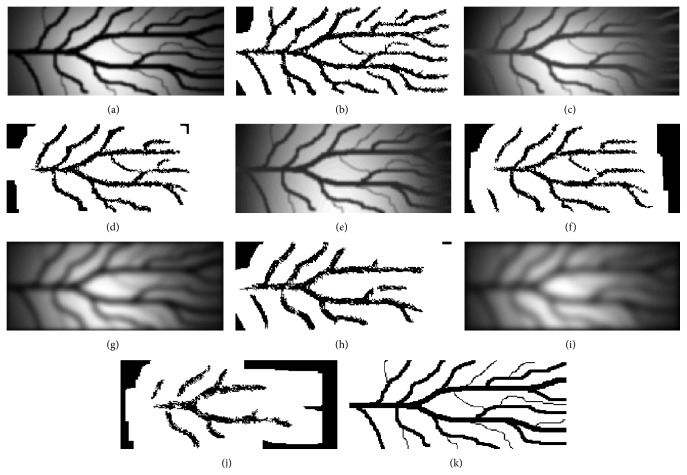
(a, c, e, g, and i) Artificial infrared images with different level of distortion, (b, d, f, h, and j) corresponding finger vein patterns extracted using the method [6, 7], and (k) manual segmentation (ground truth).

Table 2 shows mean sensitivity, specificity, and accuracy of the proposed method and the method [6, 7] for artificial infrared images with different level of distortion. These results are presented in order to indicate the robustness of the proposed method especially in low quality images.  Figure 15 shows the distorted artificial images and the corresponding finger vein patterns produced after the application of the proposed method while Figure 16 shows the distorted artificial images and the corresponding finger vein patterns produced after the application of the method presented in [6, 7].

Comparing the results presented in Tables 1 and 2, it seems that the proposed method is superior to method [6, 7] in terms of sensitivity, specificity, and accuracy for the artificial finger image database. Moreover, it is worth noting that the results of the method [6, 7] are slightly different among different executions with the same parameters and images due to the randomness introduced in the selection of the current tracking pixel and the parameters *p*_*lr*_ and *p*_*ud*_. Finally, regarding the computational complexity, the proposed method also outperforms the method [6, 7] as the number of iterations of line tracking increases.

By carefully observing the results in Table 2 and the images in Figure 16 it is obvious that the performance of the method presented in [6, 7] in low quality images is degraded and becomes unacceptable for images with high level of distortion. Thus, the proposed method outperforms line tracking method when applied to low quality image which may be produced as a result of a noncareful image acquisition setup.

As the above results show, the proposed method performs well in the majority of cases and achieves efficiently segmenting the finger vein images. However, no scientific method is perfect. Every method has also drawbacks. Thus, the main drawback of the proposed method is that its performance both in terms of segmentation accuracy and in terms of computational complexity is strongly related to the size of window neighbourhood. An appropriate selection of window neighbourhood size must be done in order to achieve meaningful results. On the other side, in case of an accurate image acquisition setup which will acquire images of specific resolution, the window size could be derived experimentally and could be then set at once.

### 4.8. Matching

In general, two methods are commonly used for matching of line-shaped patterns: structural matching [39] and template matching [40, 41]. As stated in [6, 7], structural matching requires additional extraction of feature points such as line endings and bifurcations. Since a finger vein pattern has few of these points, template matching based on comparison of pixel values is more appropriate for finger vein pattern matching. Thus, in this paper the robustness of the proposed method and the method [6, 7] for finger vein identification is evaluated by estimating the mismatch ratio between the registered and the input data for the artificial finger image database. In the matching process, the extracted finger vein pattern is converted into matching data, and these data are compared with the recorded raw data. For the case of artificial image database the ground truth data was used as recorded raw data for each image.

Mismatch ratio *R*_*m*_ is calculated to examine whether or not two sets of data have a correlation with each other. The ratio *R*_*m*_ is defined as the difference between two sets of data to be matched. *R*(*x*, *y*) and *I*(*x*, *y*) are the values at position (*x*, *y*) of the registered and input matching data, *w* and *h* are the width and height of both sets of data, *c*_*w*_ and *c*_*h*_ are the distances in which motion in the vertical and horizontal directions, respectively, is required to adjust the displacement between the two sets of data, and the template data are defined as the rectangular region within *R*(*x*, *y*) whose upper left position is *R*(*c*_*w*_, *c*_*h*_) and lower right position is *R*(*w* − *c*_*w*_, *h* − *c*_*h*_).

The value of mismatch *N*_*m*_(*s*, *t*), which is the difference between the registered and input data at the positions where *R*(*c*_*w*_, *c*_*h*_) overlaps with *I*(*s*, *t*), is defined as follows:

(21) Nm(s,t) =∑y=0h−2ch−1 ∑x=0w−2cw−1ϕIs+x,t+y,Rcw+x,ch+y,

where *w* = 212 and *h* = 87 in consideration of the finger size in the captured image, *c*_*w*_ and *c*_*h*_ are set at *c*_*w*_ = 57 and *c*_*h*_ = 38 in order to adjust the finger position in the captured image by up to about 1 cm, and *φ* in (21) is a parameter that indicates whether a pixel labeled as part of the background region and a pixel labeled as part of a vein region overlapped with each other. When *P*_1_ is defined as the pixel value of one pixel and *P*_2_ is defined as the pixel value of the other pixel, *φ* can be described as follows:

(22) $$\begin{array}{c} \phi \left(P_{1},P_{2}\right)=\left\{\begin{array}{cc} 1, & \text{if}\;\;\left|P_{1}-P_{2}\right|,\\ 0, & \text{otherwise}. \end{array}\right. \end{array}$$

The minimum value of mismatch *N*_*m*_, which is the smallest *N*_*m*_(*s*, *t*) calculated under the condition that the template overlaps with the input matching data *I*(*x*, *y*) at all positions, can be defined as follows:

(23) $$\begin{array}{c} N_{m}=\underset{0\leq s<2c_{w},0\leq t<2c_{h}}{\min}N_{m}\left(s,t\right). \end{array}$$

Using the definitions given above, the mismatch ratio *R*_*m*_ is defined as follows:

(24) Rm=Nm·∑j=t0t0+h+2ch−1∑i=s0s0+w−2cw−1ϕ(I(i,j),0)   +∑j=chh−ch−1∑i=cww−cw−1ϕ0,Ri,j−1,

where *s*_0_ and *t*_0_ are *s* and *t* such that (23) is minimized. As is shown by (24), *R*_*m*_ is described as the ratio between *N*_*m*_ and the total number of pixels that are classified as belonging to the vein region in the two data sets.

As shown in Table 3, the average mismatch ratio for the artificial finger image database, estimated using (24), is 24.65% for the proposed method while for the method [6, 7] it is 43.64%. Although database contains twenty images and the results cannot be generalized, the proposed method seems to be more appropriate for finger vein identification purposes. The extracted patterns by the proposed method have significantly lower value of mismatch ratio than those extracted by the method [6, 7].

## 5. Conclusions

In this paper an efficient finger vein pattern extraction method is presented. The proposed method is based on the minimization of the objective function of a modified Mumford Shah Model and the local application of its results. This application produces two nonsmooth images where veins located in concave regions. The two images are then combined simply by addition. Detection of concave regions is achieved via a modified local entropy thresholding technique. The preliminary segmentation result was unsatisfactory due to the presence of some outliers (misclassifications).

Thus, a final morphological postprocessing step followed in order to clean the image from the misclassifications and to produce a robust finger vein pattern. Future work includes the improvement of our imaging device in order to acquire images with less shading and noise artefacts, something that will guarantee the successful application of our method in the majority of cases. In case of images with high quality the preprocessing and/or postprocessing step can be skipped.

The experimental evaluation of the proposed method shows that it can robustly segment the finger vessel network and that the extracted finger vein pattern is appropriate for finger vein identification purposes. Finally, the proposed method is robust against strong distortions met in the acquired images.

## Figures and Tables

**Figure 17:**
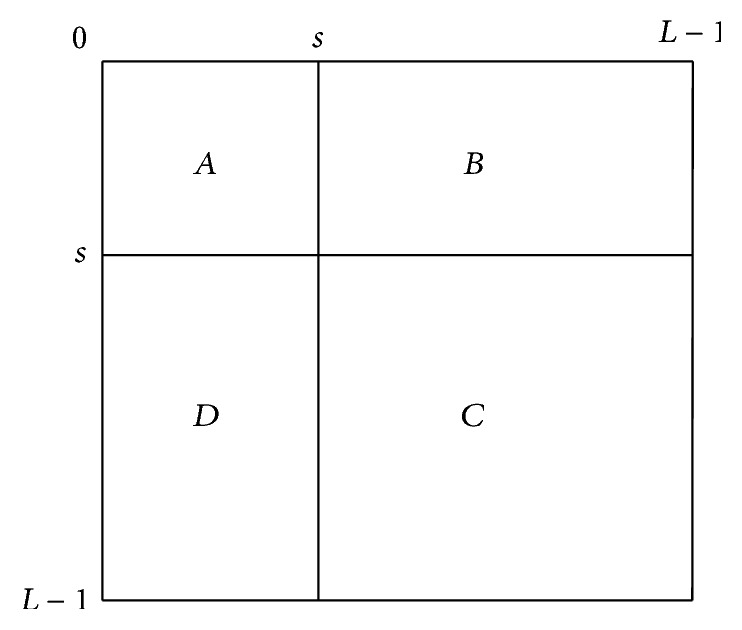
Quadrants of cooccurrence matrix.

**Table 1:** Mean sensitivity, specificity, and accuracy of the proposed method and the methods [6, 7], [11] (without preprocessing/postprocessing) and [12].

	Proposed method			Method [6, 7]		
	Sensitivity	Specificity	Accuracy	Sensitivity	Specificity	Accuracy
Mean	0.907	0.910	0.909	0.782	0.928	0.896
Standard deviation	0.073	0.018	0.027	0.075	0.037	0.041

	Method [11]			Method [12]		
	Sensitivity	Specificity	Accuracy	Sensitivity	Specificity	Accuracy

Mean	0.869	0.898	0.892	0.943	0.941	0.942
Standard deviation	0.081	0.025	0.036	0.112	0.032	0.048

**Table 2:** Mean sensitivity, specificity, and accuracy of the proposed method and the method [6, 7].

Image name	Proposed method			Method [6, 7]		
	Sensitivity	Specificity	Accuracy	Sensitivity	Specificity	Accuracy
Artificial_finger_vein.bmp	0.8779	0.9266	0.9149	0.7876	0.9047	0.8765
Artificial_finger_vein2.bmp	0.5234	0.9462	0.8444	0.5221	0.9151	0.8205
Artificial_finger_vein3.bmp	0.6144	0.9716	0.8856	0.6185	0.8395	0.7863
P1.bmp	0.8845	0.8425	0.8526	0.5477	0.9176	0.8286
P2.bmp	0.8097	0.8295	0.8247	0.4338	0.7743	0.6923
P3.bmp	0.7748	0.8139	0.8045	0.4680	0.5865	0.5580
P4.bmp	0.7349	0.8091	0.7913	0.5561	0.4013	0.4386

**Table 3:** Average mismatch ratio of the proposed method and the method [6, 7].

*Parameters* *w* = 212, *h* = 87 *c*_*w*_ = 57, *c*_*h*_ = 38	Proposed method	Method [6, 7]

Average mismatch ratio	24.65%	43.64%

## Appendix

Lemma A.1 A.1. The determinant of the symmetric matrix *A* in the form

(A.1) ANx=x−100·0−1x−10·00−1x−1·000−1x·0·····00000·x=ai,j ∣ ai,j=x,i=j−1,i=j+1∨i=j−1,  i,j=1,N0,elsewhere

can be estimated using the following recursive formula:

(A.2) $$\begin{array}{c} \left|A_{n}\left(x\right)\right|=x\left|A_{n-1}\left(x\right)\right|-\left|A_{n-2}\left(x\right)\right|,\;n=3,N,\\ \left|A_{2}\left(x\right)\right|=x^{2}-1,\;\;\left|A_{1}\left(x\right)\right|=x. \end{array}$$

Proof From the definition of matrix *A* it can be derived that

(A.3) An(x)=x−10−1x−10−1An−2(x)=xAn−1(x)+−1−10An−2(x)=xAn−1x−An−2x.

Lemma A.2 A.2. The matrix *A* determinant is an increased sequence of positive real numbers for any *x* > 2.

Proof If *x* > 2, then

(A.4) A2(x)=x2−1>3∧A1(x)= x>2⟹A2x>A1x.

If |*A*_*n*_(*x*)| > |*A*_*n*−1_(*x*)|, then

(A.5) An+1(x)=xAn(x)−An−1(x)>xAn(x)−AnxAn+1(x) =(x−1)An(x)>(2−1)An(x)An+1(x) =Anx⟹An+1x>Anx.

The induction rule leads to a sequence of positive numbers for the determinant of *A*:

(A.6) $$\begin{array}{c} \left|A_{n+1}\left(x\right)\right|>\left|A_{n}\left(x\right)\right|>\cdots >\left|A_{2}\left(x\right)\right|>\left|A_{1}\left(x\right)\right|>0. \end{array}$$

Theorem A.3 A.3. The system of linear equations is solvable for any positive value of *λ*.

Proof Based on Lemma A.2, *λ* > 0⇒*λ* + 4 > 2⇒|*A*_*n*_(*λ* + 4)| > 0.

*Statement*. Modified model produces the same results as the original model.

Proof The minimization of the objective function regarding *u*(·, ·) leads to the following equation:

(A.7) (λ+4)·u(x,y)−u(x+1,y)  −ux,y+1−ux,y−1−ux−1,y =λ·u0x,y, ∀x,y∈1,N−1,

while the minimization of the two independent objective functions (one for the second order partial derivative in the *x*-axis and one for the second directional derivative in the *y*-axis) leads to the following equations:

(A.8) (λ+2)·u(x,y)−u(x+1,y)−u(x−1,y) =λ·u0(x,y), ∀x,y∈1,N−1,

(A.9) (λ+2)·u(x,y)−u(x,y+1)−u(x,y−1) =λ·u0(x,y), ∀x,y∈1,N−1.

Adding (A.8) and (A.9) results in

(A.10) (2·λ+4)·u(x,y)−u(x+1,y)  −ux,y+1−ux,y−1−ux−1,y =2·λ·u0(x,y), ∀x,y∈1,N−1.

The only difference between (A.7) and (A.10) is that in (A.10) parameter *λ* is multiplied by a factor of 2. This makes no difference because the parameter *λ* can be arbitrarily set and has no strong impact on the final results. In other words, (A.7) and (A.10) are equivalent and produce exactly the same results if the parameter *λ* set in (A.8) and (A.9) to have the half value from the value has the corresponding parameter in (A.7).

*Local Entropy Thresholding*. The cooccurrence matrix of the image *F* = *u* − *u*_0_ is a *P* × *Q* dimensional matrix *T* = [*t*_*ij*_]_*P*×*Q*_ that gives an idea about the transition of intensities between adjacent pixels, indicating spatial structural information of an image. Depending upon the ways in which the gray level *i* follows gray level *j* different definitions of cooccurrence matrix are possible. Here, we made the cooccurrence matrix asymmetric by considering the horizontally right and vertically lower transitions. This choice has been made in order to decrease the computational cost and it is reasonable since the horizontally left and vertically upper transitions do not add more information to cooccurrence matrix. Thus, *t*_*ij*_ is defined as follows:

(A.11) tij=∑l=1P ∑k=1Quδfl,k−iδfl,k+1−j    +δfl,k−iδfl+1,k−j−1.

The probability of cooccurrence *p*_*ij*_ of gray levels *i* and *j* can therefore be written as

(A.12) $$\begin{array}{c} p_{ij}=\frac{t_{ij}}{\sum_{i}\sum_{j}t_{ij}}. \end{array}$$

If *s*, 0 ≤ *s* ≤ *L* − 1, is a threshold, then *s* can partition the cooccurrence matrix into 4 quadrants, namely, *A*, *B*, *C*, and *D* (Figure 17).

Let us define the following quantities:

(A.13) $$\begin{array}{c} P_{A}=\overset{s}{\underset{i=0}{\sum }}\;\overset{s}{\underset{j=0}{\sum }}p_{ij},\\ P_{C}=\overset{L-1}{\underset{i=s+1}{\sum }}\;\overset{L-1}{\underset{j=s+1}{\sum }}p_{ij}. \end{array}$$

From the occurrence matrix, the corresponding probabilities within each individual quadrant must sum to one. Thus, we get the following cell probabilities for different quadrants:

(A.14) PijA=pijPA=tij∑i=0s∑j=0stij, for  0≤i≤s,  0≤j≤s,   PijC=pijPC=tij∑i=s+1L−1∑j=s+1L−1tij,  for s+1≤i≤L−1, s+1≤j≤L−1.

The second order entropy of the object can be defined as

(A.15) $$\begin{array}{c} H_{A}^{\left(2\right)}\left(s\right)=-\frac{1}{2}\cdot \overset{s}{\underset{i=0}{\sum }}\;\overset{s}{\underset{j=0}{\sum }}P_{ij}^{A}\cdot \text{lo}{\text{g}}_{2}P_{ij}^{A}. \end{array}$$

Similarly, the second order entropy of the background can be written as

(A.16) $$\begin{array}{c} H_{C}^{\left(2\right)}\left(s\right)=-\frac{1}{2}\cdot \overset{L-1}{\underset{i=s+1}{\sum }}\;\overset{L-1}{\underset{j=s+1}{\sum }}P_{ij}^{C}\cdot \text{lo}{\text{g}}_{2}P_{ij}^{C}. \end{array}$$

Hence, the total second order local entropy of the object and the background can be written as

(A.17) $$\begin{array}{c} H_{T}^{\left(2\right)}\left(s\right)=H_{A}^{\left(2\right)}\left(s\right)+H_{C}^{\left(2\right)}\left(s\right). \end{array}$$

The gray level corresponding to the maximum of *H*_*T*_^(2)^(*s*) gives the optimal threshold for object background classification.

In the first modification a different definition of the cooccurrence matrix is adopted increasing the local entropy values in vein structures. As mentioned, the cooccurrence matrix of an image shows the intensity transitions between adjacent pixels. The original cooccurrence matrix is asymmetric by considering the horizontally right and vertically lower transitions. They added some jittering effect to the cooccurrence matrix that tends to keep the similar spatial structure but with much less variations; that is, *T* = [*t*_*ij*_]_*P*×*Q*_ is computed as follows:

(A.18) For  every  pixel  (l,k)  in  an  image  F   i=Fl,k,   j=Fl,k+1,   d=Fl+1,k+1,   tij=tid+1.

One may wonder whether the modified cooccurrence matrix still will represent the original spatial structure. Actually, considering a smooth area in an image where *j* and *d* should be very close or identical, the above computation in (A.18) implicitly introduces certain smoothing effect and adds some structured noise to the cooccurrence matrix. The two matrices still share a similar structure that is important for the valid thresholding result. Also, the latter one has larger entropy with a much smaller standard deviation, which is more desirable for local entropy thresholding.

Secondly, they considered the sparse foreground in selecting the optimal threshold. The original threshold selection criterion aims to maximize the local entropy of foreground and background in a gray scale image without considering the small proportion of foreground. Therefore, they proposed selecting the optimal threshold that maximizes the local entropy of the binarized image that indicates the foreground/background ratio. The larger the local entropy the more balanced the ratio between foreground and background in the binary image is.

## References

[1] Park G. T., Im S. K., Choi H. S. A person identification algorithm utilizing hand vein pattern.

[2] Hong D. U., Im S. K., Choi H. S. (1999). Implementation of real time system for personal identification algorithm utilizing hand vein pattern. *Proceedings of the IEEK Fall Conference*.

[3] Im S.-K., Park H.-M., Kim Y.-W. (2001). An biometric identification system by extracting hand vein patterns. *Journal of the Korean Physical Society*.

[4] Im S. K., Park H. M., Kim S. W., Chung C. K., Choi H. S. Improved vein pattern extracting algorithm and its implementation.

[5] Im S. K., Choi H. S., Kim S.-W. (2003). A direction-based vascular pattern extraction algorithm for hand vascular pattern verification. *ETRI Journal*.

[6] Miura N., Nagasaka A., Miyatake T. (2004). Feature extraction of finger-vein patterns based on repeated line tracking and its application to personal identification. *Machine Vision and Applications*.

[7] Miura N., Nagasaka A., Miyatake T. (2004). Feature extraction of finger vein patterns based on iterative line tracking and its application to personal identification. *Systems and Computers in Japan*.

[8] Vlachos M., Dermatas E. A finger vein pattern extraction algorithm based on filtering in multiple directions.

[9] Tanaka T., Kubo N. Biometric authentication by hand vein patterns.

[10] Ding Y., Zhuang D., Wang K. A study of hand vein recognition method.

[11] Vlachos M., Dermatas E. (2013). A segmentation method using multiscale and multidirectional matched filters based on dyadic wavelet transform for finger vein pattern extraction. *Electrical Engineering Research*.

[12] Vlachos M., Dermatas E. (2009). Finger vein segmentation and centerlines extraction in infrared images. *Advanced Topics in Scattering Theory and Biomedical Engineering: Proceedings of the 9th International Workshop on Mathematical Methods on Scattering Theory and Biomedical Engineering, Patras, Greece, 9–11 October 2009*.

[13] Yan W., Xie J., Li P., Liu T., Guo X. (2013). An algorithm for vein matching based on log-polar transform. *Smart Computing Review*.

[14] Liu T., Xie J., Lu H., Yan W., Li P. (2013). Finger vein representation by modified binary tree model. *The Smart Computing Review*.

[15] Yang G. P., Xiao R. Y., Yin Y. L., Yang L. (2013). Finger vein recognition based on personalized weight maps. *Sensors*.

[16] Yang J. F., Shi Y. H. (2012). Finger-vein ROI localization and vein ridge enhancement. *Pattern Recognition Letters*.

[17] Yang J., Shi Y., Yang J. (2011). Personal identification based on finger-vein features. *Computers in Human Behavior*.

[18] Song W., Kim T., Kim H. C., Choi J. H., Kong H.-J., Lee S.-R. (2011). A finger-vein verification system using mean curvature. *Pattern Recognition Letters*.

[19] Paquit V., Price J. R., Seulin R. Near-infrared imaging and structured light ranging for automatic catheter insertion.

[20] Zeman H. D., Lovhoiden G., Vrancken C. The clinical evaluation of vein contrast enhancement.

[21] Zeman H. D., Lovhoiden G., Vrancken C. Prototype vein contrast enhancer.

[22] Chan T. F., Shen J. (2005). *Image Processing and Analysis: Variational, PDE, Wavelet, and Stochastic Methods*.

[23] Esedoglu S., Shen J. (2002). Digital inpainting based on the Mumford-Shah-Euler image model. *European Journal of Applied Mathematics*.

[24] Vo-Dinh T. (2003). *Biomedical Photonics Handbook*.

[25] Lee E. C., Lee H. C., Park K. R. (2009). Finger vein recognition using minutia-based alignment and local binary pattern-based feature extraction. *International Journal of Imaging Systems and Technology*.

[26] Chaudhuri S., Chatterjee S., Katz N., Nelson M., Goldbaum M. (1989). Detection of blood vessels in retinal images using two-dimensional matched filters. *IEEE Transactions on Medical Imaging*.

[27] Mumford D., Shah J. (1989). Optimal approximations by piecewise smooth functions and associated variational problems. *Communications on Pure and Applied Mathematics*.

[28] Ran R.-S., Huang T.-Z. (2006). The inverses of block tridiagonal matrices. *Applied Mathematics and Computation*.

[29] El-Mikkawy M. E. A. (2004). On the inverse of a general tridiagonal matrix. *Applied Mathematics and Computation*.

[30] da Fonseca C. M., Petronilho J. (2001). Explicit inverses of some tridiagonal matrices. *Linear Algebra and Its Applications*.

[31] Meurant G. (1992). A review on the inverse of symmetric tridiagonal and block tridiagonal matrices. *SIAM Journal on Matrix Analysis and Applications*.

[32] El-Mikkawy M. E. A. (2003). A note on a three-term recurrence for a tridiagonal matrix. *Applied Mathematics and Computation*.

[33] Golub G. H., van Loan C. F. (2001). *Matrix Computation*.

[34] Ding L. J. (1997). *Numerical Computing Method*.

[35] Chanwimaluang T., Fan G. An efficient algorithm for extraction of anatomical structures in retinal images.

[36] Pal N. R., Pal S. K. (1989). Entropic thresholding. *Signal Processing*.

[37] Pal N. R., Pal S. K. http://www.vcipl.okstate.edu/Publications/TITBcorrection.pdf.

[38] Gongalez R., Woods R., Eddins S. (2003). *Digital Image Processing Using Matlab*.

[39] Weiwei Z., Yangsheng W. Core-based structure matching algorithm of fingerprint verification.

[40] Nagao M. (1983). *Methods of Image Pattern Recognition*.

[41] Jain A. K., Duin R. P. W., Mao J. (2000). Statistical pattern recognition: a review. *IEEE Transactions on Pattern Analysis and Machine Intelligence*.

